# Women's experiences of care after stillbirth and obstetric fistula: A phenomenological study in Kenya

**DOI:** 10.1111/hex.13841

**Published:** 2023-08-17

**Authors:** Anne Nendela, Sarah Farrell, Sabina Wakasiaka, Tracey Mills, Weston Khisa, Grace Omoni, Tina Lavender

**Affiliations:** ^1^ Lugina Africa Midwives' Research Network Kenyatta National Hospital Nairobi Kenya; ^2^ Department of International Public Health Centre for Childbirth, Women's and Newborn Health, Liverpool School of Tropical Medicine Liverpool UK; ^3^ School of Nursing Sciences University of Nairobi Nairobi Kenya; ^4^ Reproductive Health Department Kenyatta National Hospital Nairobi Kenya

**Keywords:** global health, obstetric fistula, stillbirth, sub‐Saharan Africa, women's experiences

## Abstract

**Background:**

Stillbirth and (obstetric) fistula are traumatic life events, commonly experienced together following an obstructed labour in low‐ and middle‐income countries with limited access to maternity care. Few studies have explored women's experiences of the combined trauma of stillbirth and fistula.

**Aim:**

To explore the lived experiences of women following stillbirth and fistula.

**Methods:**

Qualitative, guided by Heideggerian phenomenology. Twenty women who had experienced a stillbirth were interviewed while attending a specialist Hospital fistula service in urban Kenya. Data were analysed following Van Manen's reflexive approach.

**Results:**

Three main themes summarised participants' experiences: ‘Treated like an alien’ reflected the isolation and stigma felt by women. The additive and multiplying impacts of stillbirth and fistula and the ways in which women coped with their situations were summarised in ‘Shattered dreams’. The impact of beliefs and practices of women and those around them were encapsulated in ‘It was not written on my forehead’.

**Conclusion:**

The distress women experienced following the death of a baby was intensified by the development of a fistula. Health professionals lacked an understanding of the pathophysiology and identification of fistula and its association with stillbirth. Women were isolated as they were stigmatised and blamed for both conditions. Difficulty accessing follow‐up care meant that women suffered for long periods while living with a constant reminder of their baby's death. Cultural beliefs, faith and family support affected women's resilience, mental health and recovery. Specialist services, staff training and inclusive policies are needed to improve knowledge and awareness and enhance women's experiences.

**Patient or Public Contribution:**

A Community Engagement and Involvement group of bereaved mothers with lived experience of stillbirth and neonatal death assisted with the review of the study protocol, participant‐facing materials and confirmation of findings.

## 1 INTRODUCTION

Two million stillbirths occur globally every year, of which 84% occur in low‐ and lower middle‐income countries.^1^ In 2014, the World Health Assembly set a target for reducing the stillbirth rates to 12 or fewer stillbirths per 1000 births by 2035.^2^ Kenya has introduced the National Hospital Insurance Fund and free maternity care to improve access to maternity services^3^; however, the stillbirth rate has remained static, with estimations of 19.67 per 1000 births in 2013 and 19.75 per 1000 births in 2019.^4^ This is similar to other sub‐Saharan countries where stillbirths have stagnated or even increased due to population growth.^1^ A previous study has shown a link between obstetric fistula and stillbirth, highlighting that a lack of access to antenatal care and to quality intrapartum obstetric care are risk factors for both.^5^ Obstetric fistula is an abnormal connection between the vagina and the rectum and/or the bladder which usually develops after prolonged and obstructed labour and leads to continuous urinary or faecal incontinence.^6^ The majority occur in sub‐Saharan Africa and South Asia,^6^ with 3000 new cases reported annually in Kenya, which equates to an incidence of 1 in 1000 births.^7^

Although the impacts of the death of a baby before or during birth have recently received greater attention globally,^8^ the experiences of women affected by the ‘double burden of tragedy’ of fistula and stillbirth^5^ have not been widely examined. In sub‐Saharan Africa, stillbirth and fistula are commonly attributed to spirits, sin or ‘curses’ which portion blame on the women or their families.^9^, ^10^ Similarly, marital conflicts or divorce may occur after a stillbirth as women have not met the expectation of childbearing, or because of stigma associated with uncontrolled urine from a fistula.^11^ It is not known if stigmatisation or rejection is different for women who have suffered both. It has been estimated that 4.2 million women are living with depression following an experience of stillbirth^12^ yet this may be amplified further for women who have ongoing incontinence and poor physical health.

### 1.1 Aim

This study aimed to explore the lived experiences of women following stillbirth and fistula in Kenya. Few studies have considered the impact of this dual trauma but understanding women's experiences will help direct future research and improvements in care provision.

## 2 METHODOLOGY AND METHODS

### 2.1 Study design

This qualitative study was guided by Heideggerian phenomenology,^13^ and sought to capture the lived experiences of women who had experienced the dual trauma of stillbirth and fistula. The hermeneutic methodology allows understanding of experiences as told by the women themselves as the researcher uses a reflexive approach moving between the parts and whole of the data to achieve a more detailed and nuanced understanding.^14^ The primary researcher, a Kenyan midwife with experience in providing care to families following a stillbirth, designed and facilitated the study with support from two Kenyan academic health professionals and two UK‐based academic midwives. A Community Engagement and Involvement (CEI) group, contributed to the study design, recruitment and consent strategy, data collection processes and data interpretation.

### 2.2 Setting and participants

The study was conducted in a large urban teaching hospital in Kenya. Women who were at least 18 years old were eligible if they had experienced a stillbirth within the previous 3 years and were subsequently diagnosed with a fistula. Women had to understand the study information and be willing to provide informed consent (written or thumbprint). A purposive sample of up to 20 women was estimated, based on achieving data adequacy.^15^

Eligible women were approached by healthcare professionals in the fistula repair centre during their admission for surgery. The study was introduced and those interested completed a ‘consent to contact’ form. Women were given further verbal and written information about the study and a convenient time and place was arranged for the interview. The final sample included 20 women who met the inclusion criteria and were willing to participate.

### 2.3 Data collection

One‐to‐one interviews were conducted between June 2019 and September 2019 by the primary researcher, a midwife experienced in conducting interviews with bereaved parents, who received additional training in research methods. All participants chose to be interviewed in Kiswahili, in a private counselling room within the hospital, facilitated by the researcher as a native speaker.

Demographic and clinical data were collected using a researcher‐designed questionnaire. In keeping with a phenomenological approach, an open respondent‐led interviewing style was employed, supported by an interview topic guide. Broad opening questions such as ‘can you tell me about the care you received after your baby died?’ were used, with minimal additional prompts to ensure sufficient depth. The researcher followed the woman's lead in referring to the stillborn baby during the interview, for example, using the baby's name in the interview if the woman wished. The researcher reflected the main points back to the participant at the end of the interview, to ensure accurate interpretation by the researcher and reliability of the data.^16^ Interviews were audio‐recorded, transcribed verbatim and translated into English. Contemporaneous field notes captured nuances, and reflexive diaries were completed immediately after every interview documenting interview dynamic, learning points and emergent themes.

Some women became emotional during interviews as they recalled their traumatic experiences. The sensitivity of the topic area was recognised throughout the research process, and the research midwife used a distress policy to clarify whether women wished to continue with the interview, allowing time for women to pause or reflect where necessary. The interviewer, an experienced midwife, was able to debrief women after each interview, and no onward referral was necessary. However, contact details were provided to all women, should they wish to receive subsequent counselling. All women chose to continue with the interviews, and many expressed their gratitude for having an opportunity to discuss their experiences.

### 2.4 Analysis

Data analysis was guided by the principle of transforming lived experience into a textual expression of its meaning using Van Manen's reflexive three‐stage approach.^17^ First, the anonymised narratives were considered as a whole, reading and rereading transcripts to achieve familiarisation.^18^ Second, significant statements related to the experience of care after stillbirth and fistula were highlighted in individual narratives with the use of memos documenting explanations. Next, a detailed approach involved line‐by‐line consideration of narratives and clustering of similarities. Identification of subthemes and main themes resulted from a process in line with Heidegger's key concept of the hermeneutic circle where the researcher moves between the whole and the parts of the data using a cyclical approach.^14^ Analysis was conducted by the primary researcher with support from academic supervisors, before confirming the overall interpretation and definition of the essential nature of the studied phenomenon with input from the wider research and CEI team.

## 3 FINDINGS

Interviews were conducted with 20 women (Table 1). Four stillbirths occurred in the antenatal period, with 16 occurring intrapartum. Five women had experienced a prior stillbirth and nine of the women had living children. Nine of the women were married, five were divorced or separated, five were single and one widowed. The average age of the women was 30.7 years with an average of 10.05 years of education.

**Table 1:** Participant characteristics.

Pseudonym		Age	Marital status	Years of school	Occupation before fistula	Gestation at birth	Type of birth	Type of stillbirth	Number of living children	Number of previous stillbirths
Nicole		34	Divorced	13	Waitress	32	Caesarean	Antenatal	1	0
Sharon		20	Married	13	Nursery teacher	40	Spontaneous vaginal birth	Antenatal	0	0
Kendall		34	Married	13	Tailor	34	Spontaneous vaginal birth	Antenatal	4	0
Laura		32	Divorced	12	None	34	Spontaneous vaginal birth	Intrapartum	2	0
June		25	Married	9	None	38	Caesarean	Intrapartum	0	1
Eleanor		43	Single	9	None	28	Spontaneous vaginal birth	Intrapartum	0	1
April		19	Single	13	None	40	Spontaneous vaginal birth	Intrapartum	0	0
Suzanne		42	Divorced	9	Shop assistant	40	Caesarean	Intrapartum	0	0
Anita		51	Married	9	None	38	Cesarean	Intrapartum	2	9
Kelly		21	Married	12	None	32	Caesarean	Intrapartum	2	0
Kerri		19	Single	11	House help	42	Spontaneous vaginal birth	Intrapartum	0	0
Sheralee		42	Separated	7	None	28	Spontaneous vaginal birth	Intrapartum	2	0
Stephan		27	Single	3	Farmer	32	Caesarean	Intrapartum	2	0
Alysha		26	Married	8	None	28	Caesarean	Intrapartum	0	5
Nick		35	Divorced	10	None	38	Caesarean	Intrapartum	0	0
Muriel		44	Widowed	7	Farmer	44	Caesarean	Intrapartum	5	3
Megan		18	Single	7	Student	36	Vacuum	Intrapartum	0	0
Lizzie		20	Married	13	Housewife	41	Caesarean	Intrapartum	0	0
Maryanne		28	Married	10	Farmer	38	Caesarean	Intrapartum	0	0
Muire		34	Married	13	Business	32	Caesarean	Antenatal	1	0

Three main themes summarised the interpretation of participants' experiences following the stillbirth and fistula, with the theme titles taken from verbatim quotes. Theme 1 ‘treated like an alien’ summarises feelings of less favourable reception and marginalisation following stillbirth and fistula, with the subthemes of being ‘unaware and uninformed’ and ‘social and care inequalities’. The extreme sense of loss felt by mothers is described in theme 2 ‘shattered dreams’ and is summarised in the subthemes ‘coping with stillbirth and fistula’ and ‘double distress and despair’. The resilience of women is reflected in theme 3 ‘it was not written on my forehead’, with the subtheme ‘beliefs and practices’ reflecting how the beliefs and practices of women and those around them impacted them in positive and negative ways.

### 3.1 Theme 1: ‘Treated like an alien’

#### 3.1.1 Isolation and rejection

Women were often treated negatively and marginalised following stillbirth, before the fistula was even realised. Cultural beliefs that associated stillbirth with bad omens affected the way women were regarded, even in the healthcare setting. Decisions about whether the mother saw her baby and plans for burial were made by health professionals or family members. One woman, who was unable to see her baby, encapsulated the views of other participants when she described how ‘they treated me like an alien’:

No, I didn't see the baby. The only person who saw the baby was my partner. I would have loved to see my baby, but the nurses and doctors didn't allow me. They treated me like an alien, yet it was my own blood and flesh”… she [her mother] didn't treat me well, treated me like I was not her daughter…an outcast. …even my brothers denied me. I struggled until I delivered a baby, and I reached out to my baby's father, the man tortured me and would burn me with a cigarette on my nose… [Laura]

Stillbirth was often linked to the presence of evil spirits, which led to women being stigmatised or blamed for the death of the baby. While almost half of the women remained married and almost half had other children, many of the women described being rejected by a partner, members of their family or by community members. Some spouses abandoned women before they left the hospital, a period of extreme physical and emotional vulnerability. June, who was extremely unwell after birthing her stillborn baby recalled:

…after the operation [caesarean section], I never heard from him [her husband]. I heard he got married. …after seeing me he assumed that I was not going to make it and he went on starting a new life. [June]

The subsequent development of a fistula increased the isolation and abandonment, as cultural beliefs labelled women as cursed or to blame for their incontinence, and because they were considered ‘unhygienic’ or ‘smelly’. The debilitation caused by the fistula, combined with this rejection, further affected women's mental health, and led to some experiencing suicidal thoughts. While some women were treated as outcasts and ignored by those previously close to them, some were physically and verbally abused or made to feel worthless by the actions of others. This humiliation resulted in some women choosing to leave home, such as Suzanne, whose spouse refused to eat food she prepared suggesting it had been tainted by the fistula:

I left him… Imagine someone walking home and finds that you have prepared food and pours it, what does that mean? [Suzanne]

#### 3.1.2 Unaware and uninformed

Following the stillbirth, women were frustrated by the lack of information provided to them and felt that their questions were not answered. Sometimes they were deceived and ‘left in the dark’ for long periods, increasing a sense of alienation. Evasiveness by health professionals meant that some women only received news of the baby's death through overheard conversations:

I remember cleaners discussing… I was listening and one of them said a lady who was in the theatre the day before yesterday, her baby has died…A lady came in a bad state in an ambulance. Immediately I knew they were talking about me and my baby. I started crying. [June]

A lack of knowledge and understanding of what caused the stillbirth and fistula made the women feel disadvantaged and insignificant and impacted their ability to process the experience of the death of their baby and ongoing health complications. They described poor postnatal care practices where the diagnosis of fistula was delayed, having had incontinence ignored or dismissed by health professionals who expected incontinence to resolve:

I would wake up and find the bed wet and I went to the doctor and the doctor told me that if the uterus has been removed then that is something normal and the liquid that came out had some medicinal smell. I went back to him again and he told me it would stop on its own and I should give it time. [Laura]

#### 3.1.3 Social and care inequalities

Women's income was affected by the stigma of stillbirth and fistula and by their ongoing health concerns. Employment was sometimes terminated as women were considered unclean and unable to cook or clean for other people. Others were unable to work due to incontinence or leg pain and difficulty walking caused by nerve damage. The inability to purchase basic hygiene necessities such as incontinence pads or soap increased women's despair leading to some having suicidal thoughts:

I felt really bad, and I keep asking myself, why do I have to suffer this much? It's even better dying and leaving this world than suffering the way I have. I cannot even work due to the current situation that I have because of my inability to control bowel movements, and can you even work in anyone's house in this condition? You cannot even wash for anyone's clothes [washed clothes for a livelihood], who will even allow you? I don't even go anywhere when I am at home, I just stay in the house, and I am telling you it's serious. [Anita]

Financial constraints impacted access to fistula repair as there are few specialist clinics, often far from the women's homes. The difficulty in accessing help left women feeling helpless and amplified their loneliness. When women identified fistula services and travelled long distances in the hope of finding help, the high cost of repair services remained a barrier for some:

I stayed for a month… and came back to the hospital, the doctor told me that he cannot handle that condition at [Name of Hospital], I should go to [Name of Hospital], they were asking for three hundred thousand Kenya shillings [approximately £2000] and we did not have that money and we went home. [Eleanor]

Even when women could access care for the fistula, information provided by the hospital on speciality clinics was confusing, resulting in women attending clinics that could not cater for their needs. This resulted in some women attending multiple times before being treated. The women felt frustrated by the system and experienced additional emotional and financial stress:

…I had been given an appointment to the clinic at the hospital after two months and they told me they do not treat my sickness, they referred me to [name of hospital] …on a Friday to [name of the clinic], they told us that the clinic days for obstetric fistula is Monday. After coming back on Monday, we thought I was going to be admitted but we found that you have to go on a system and they take four women every Monday, …had to try our luck and if you get late you still do not get attended… [Nicole]

### 3.2 Theme 2: ‘Shattered dreams’

Societal expectations in Kenya, like many parts of Africa, is that couples should have children to make their family complete and that the identity of women is related to them becoming a mother. Laura expressed:

…when I went for my ante‐natal, had an ultrasound and they told me am carrying a boy …it was my dream one day I would have a boy child. [Laura]

Women described their desire and expectation of having a live baby and recovering well after birth. Women did not expect adverse outcomes for themselves, despite the incidence of stillbirth and fistula in Kenya. The stillbirth and fistula were particularly surprising to women following a straightforward pregnancy where they had attended antenatal care and they felt their dreams had been shattered.

#### 3.2.1 Coping with the stillbirth and fistula

Stigma and isolation meant women frequently grieved alone and struggled to meet their basic needs through lost income and non‐existent family provision. Women felt unable to participate in normal community activities such as shopping, visiting friends or going to church because of the perceived stigma. This caused them ongoing sadness and loss:

…in church I cannot take my offering, I send someone …I walk with a bag containing my clothes because I might need to change anytime. …If a Neighbour asks for help to attend guests, it is I don't feel like going because you will hear people say why did so and so come and she urinates, …she will touch the utensils, she should have stayed away and let other people do the work, when you hear such comments it's painful. [Eleanor]

Some women received psychological support from family or friends, which helped them as they processed their grief. For some it was helpful to be reminded that they could have another baby in the future and be thankful that their own life had not been lost. Practical support from family such as provision of adult diapers, or to seek help for the fistula also contributed positively to women's mental health and recovery:

Yes, my sister did and even my cousin called me to inform me [about the fistula camp], but I had already come here, and she wished me well. And remember that is a male cousin who was discussing that with me because he wants me to recover and go back to being myself because this disease causes you a lot of discomforts…All of them were supportive of both my family and even my husband's family were very supportive. And they encouraged me until I started to feel better. [Suzanne]

#### 3.2.2 Double distress and despair

Stillbirth and the development of a fistula both contributed to women's distress and despair. Initially, the stillbirth was highly distressing, especially before the fistula was identified, and being cared for on a postnatal ward with other babies exacerbated this. Women felt isolated in their grief as they were unable to care for and breastfeed their own baby like other mothers. The pain of the death was exacerbated by hearing other babies:

I remember it was not easy because in the wards you could hear the other mothers who had babies who were alive crying during the night. And then you would even wake up and touch the beddings thinking it was your baby who was crying but then I came to realize that the reality was that I actually don't have any baby. [Nicole]

Some women articulated that the stillbirth and fistula were equally distressing to them, and that they felt the ongoing pain of both. The death of the baby was seen as an enduring loss of the child's contribution to the household:

Both are bad [fistula and stillbirth], my baby was equally important because I need a baby to have around and to send when I need certain things done…… so losing my child was equally painful. [Kelly]

Women described how their ongoing emotional distress and diminished sense of self‐worth were compounded by the physical effects of the fistula. Incontinence impacted the way they dressed, going out and socialising:

It is not the [stillborn] boy now because the fistula is really stressing. I cannot inter‐ mingle, cannot go to church and even visit a friend because you will think when you wake up the urine will be all over the seat. You start wearing incontinence pants. It's hectic to have fistula. [Nicole]

As time passed it was clear that for some women the fistula had become a greater problem, a constant embarrassment, or ‘punishment’ that took away their happiness and social interactions and restricted their life. Kelly later said:

…losing my child, it is not a big problem; I can get another one, but this fistula has brought many challenges to my life. [Kelly]

### 3.3 Theme 3: ‘It was not written on my forehead’

Despite the traumatic experiences, many of the women demonstrated resilience that enabled them to continue with their lives. Comfort was found in their problems being hidden from some people. They were optimistic they would not be defined by these experiences as they were not ‘written on their foreheads’ and that after the fistula surgery they would resume daily activities:

My child died but it was not written on my forehead that I had lost a baby. I quickly forgot about it after consolation from friends and my daughter. [Laura]

#### 3.3.1 Beliefs and practices

Women's experiences, coping and adjustment were affected by the beliefs and practices they encountered and the extent to which they agreed with them. The prevalent belief held within the women's communities was that there was an association between stillbirth or fistula and familial or individual curses:

The fistula he [her husband] said it was as a result of the curse from my family and that really hurt me a lot. [Laura]

These beliefs were not always shared by the women themselves, but resulted in significant emotional hurt and feeling rejected by their community:

They [community members] said I am an outcast that is why such things keep happening to me, and others were advising me to go to a traditional healer…I don't believe that and the fact I took long to recover some people were convinced it was an evil spirit. [June]

I was just normal and then they heard that I had fistula and so they started asking who had bewitched me. …some even naming names. [Lizzie]

Some women were influenced by beliefs within their family or community, believing that the stillbirth and fistula were direct consequences of actions that they had taken or that they were being punished for, and so blamed themselves. June describes:

…punishment for not listening to my mum, she kept telling me to go to school and forget about boys, but I never listened to her. Maybe it was a punishment and wake‐up call for me …. to think of my future. [June]

Religious beliefs were a particular source of comfort, with several women alluding to ‘God's will’. Reassurance from church members helped some women to remain positive for the future as they hoped for a resolution to the fistula. Some found their faith in God enabled them to be an encouragement to family members:

He [Husband] has slightly recovered but what has even made him feel sadder [than losing a baby] was him thinking about this condition that I have developed if I was really going to recover. And I told him God is more powerful than any human being. [Lizzie]

## 4 DISCUSSION

This study explored the lived experiences of women who had experienced the combined trauma of stillbirth and fistula using a phenomenological approach. In‐depth interviews allowed insight into women's journeys from the time of the stillbirth and fistula occurring until the hospital admission to receive restorative treatment. The experiences of women have been reflected in three main themes. Women described being ‘treated like an alien’ due to the poor postnatal care received and the negative treatment by their family or community when they arrived home. ‘Shattered dreams’ encapsulated the feeling of despair, which started with the death of the baby, and extended to the loss of family life and living with a chronic health condition. The resilience of women was demonstrated in ‘it was not written on my forehead’, acknowledging that despite traditional beliefs and customs affecting their treatment and emotional recovery, they had found ways of coping to help them survive.

The lack of information, evasiveness, or misinformation from health professionals following stillbirth and fistula created anxiety in women and made them feel insignificant. These findings resonate with a previous study in Uganda, whereby healthcare providers were described as unsupportive following stillbirth, not providing consolation or an explanation about the cause of the stillbirth, with some mothers discovering the death themselves.^19^ Similarly, a study of lived experiences in Malawi found women had little knowledge and understanding of fistula, unless a family member was previously affected, which led to misunderstanding and misattribution of the cause as the caesarean section surgery, their husband's unfaithfulness or witchcraft.^20^ Being ‘left in the dark’, being unable to understand what caused the death of a baby due to a lack of explanation from staff, also impacted on Kenyan and Ugandan parents’ experiences, with delays and deceit surrounding acknowledgement of the death adding to their distress.^21^ The need for education of health professionals and ongoing research to improve care has been identified, to reduce anxiety and help women understand the aetiology of their problems.

Rejection and isolation were reported by some women because of cultural beliefs about the causes of stillbirth and fistula, and others were treated with abuse. Women reported that this worsened after the fistula had been identified, similar to another study in Kenya.^22^ This supports wider findings from studies of women's experiences following obstetric fistula, such as a recent phenomenological study in Ethiopia which found the ‘painful social life’, caused by discrimination and stigma associated with having leakage of urine, resulted in feelings of isolation as women were unable to participate in social activity.^23^ While many participants, in our study, grieved in isolation, some received emotional and practical help from family or church friends. Many women took comfort in their faith which helped them deal with the loss of their baby and the consequences of the fistula. A study in Uganda also found the role of faith to be significant in recovery after stillbirth, helping women find comfort and hope for the future.^24^ However, despite women finding comfort from family or in their faith, the continuous leakage of urine prevented attendance at social gatherings or at church, similar to women interviewed about their lived experiences of fistula in Tanzania.^25^ The social isolation also led to financial difficulty as women had employment terminated, were unable to work or lost financial support from family. These narratives reflect those of a multi‐site study in Uganda and Kenya,^9^ and emphasise the need for community education to directly address the misconceptions around causes of stillbirth and fistula. This would be beneficial for women as increased social support has been shown to correlate with lower levels of depression following obstetric fistula in Tanzania.^26^

Accessing specialist care for fistula repair was difficult for women in this study due to the transportation and treatment costs, and the confusion regarding the hospital system. Changole, Thorsen and Kafulafula^20^ believed that stigmatisation acts as a further barrier to seeking help. Some women, in our study, described how they hid their trauma from those around them to minimise rejection and isolation. However, this may not always be beneficial as the secrecy surrounding fistula was a barrier to Kenyan women seeking or being offered treatment in another study.^27^ Referral and access to fistula centres for restorative surgery must be increased, enabling women to get prompt help regardless of their ability to pay.

Women did not expect poor outcomes and had to adjust and cope after their ‘dreams had been shattered’. Similarly, in Uganda, parents described the pain and confusion associated with stillbirth, feeling heartbroken returning home without their baby, and of coping with the loss of future dreams of the child growing up and supporting the family.^19^ Women initiated coping mechanisms to conceal the loss or leakage associated with fistula, isolating away from other people or relocating to a new place, like what women described in Malawi.^28^ Poor mental health was commonly reported, starting on the postnatal ward and becoming worse following the fistula diagnosis. Similarly, other studies have highlighted the damaging effect of fistula on women's mental health.^29^, ^30^ A scoping review from Nigeria about the psychosocial impact of fistula and available support, found that little research has considered psychosocial interventions despite a clear evidence base indicating severe psychological outcomes for women with fistula.^31^ However, one of the included studies ‘reported a rehabilitation program offered to the affected women which made them resilient and hopeful’.^31^ Specialist mental health services are not yet widely available and accessible to women in Kenya, but this must be addressed in line with the World Health Organisation recommendation that psychosocial support must be included in care of women following obstetric fistula.^32^

### 4.1 Strengths and limitations

Although care provision for women following stillbirth or fistula has been previously studied, uniquely, this research explored women's experiences of both, contributing additional insight and nuanced understanding. This study included women who had attended a fistula treatment centre in one urban site in Kenya; findings are likely to be transferrable to other settings, but further research is required to confirm this. The views and experiences of women living in rural areas without access to restorative surgery, or who may have been affected by fistula for longer than 3 years, as included in this study, may provide different views.

## 5 CONCLUSION AND RECOMMENDATIONS

Women, in this study, demonstrate the negative physical, social, and psychological impact of stillbirth and fistula, illuminating the amplification of effect when experiencing both. It is important that maternity care remains free in Kenya and that accessibility is improved so that women do not delay in seeking maternity care. Efforts must also continue to improve maternity care access and provision in Kenya to reduce stillbirths and eradicate the incidence of fistula. For women that have experienced stillbirth and fistula, there is currently little support available in Kenya, as in many other low‐ and middle‐income countries.

Staff training is needed to address health workers' knowledge of the unique vulnerability of women affected by this combination of poor outcomes. Improvements within the health system need to ensure prompt recognition, accurate information giving and optimal follow‐up care. Standards and guidelines to support health professionals must be developed to outline optimal and compassionate care. Education of new nurses and midwives must include caring for women following stillbirth, including understanding the relationship between stillbirth and fistula.

Immediate and ongoing psychological support for women must be prioritised. Accessibility and referral mechanisms must be addressed to enable all women to access fistula repair including those from rural or low socioeconomic backgrounds. Education is also needed about the causes of stillbirth and fistula to dispel the myths and stigma and to help families and communities understand the needs of women.

## AUTHOR CONTRIBUTIONS

**Anne Nendela**: Conceptualisation; methodology, data collection; data analysis; writing—review and editing. **Sarah Farrell**: Data analysis; writing—original draft. **Sabina Wakasiaka**: Data collection; data analysis. **Tracey Mills**: Supervision; conceptualisation; data analysis; writing—review and editing. **Weston Khisa**: Data collection. **Grace Omoni**: Conceptualisation. **Tina Lavender**: Supervision; conceptualisation; data analysis; writing—review and editing.

## CONFLICT OF INTEREST STATEMENT

The authors declare no conflict of interest.

## ETHICS STATEMENT

The study was approved by the Research and Ethics Committees of the University of Manchester (2017‐0233‐4462) on 22 December 2017 and the University of Nairobi (P240/05/2017) on 18 January 2019. Administrative clearance was provided by the included health facilities (P788/11/2018). Written informed consent was obtained from all participants, including for the use of anonymised verbatim quotes. Relevant guidance and regulations were followed at all stages of the research process, including recruitment, data collection and data analysis. Pseudonyms were chosen by participants, and these were used during transcription and in published verbatim quotes.

## Data Availability

The deidentified data sets used and/or analysed during the current study are not publicly archived but are available from the corresponding author at a reasonable request.
